# A Lyophilizable Nanoparticle Anthrax Vaccine Targeting the Loop-Neutralizing Determinant in Protective Antigen from *Bacillus anthracis*

**DOI:** 10.3390/microorganisms13081878

**Published:** 2025-08-12

**Authors:** Jon Oscherwitz, Kemp Cease, David Milich, Tod Merkel, Thomas Braun, Fen Yu, David C. Whitacre

**Affiliations:** 1 Division of Hematology-Oncology, Department of Internal Medicine, University of Michigan Medical School, Ann Arbor, MI 48105, USAfenyu@umich.edu (F.Y.); 2 Veterans Administration Ann Arbor Healthcare System, 2215 Fuller Road, Ann Arbor, MI 48105, USA; 3 VLP Biotech, Inc., 3030 Bunker Hill St., Ste 117D, San Diego, CA 92109, USA; dmilich@vlp-biotech.com (D.M.); dwhitacre@vlp-biotech.com (D.C.W.); 4 Division of Bacterial, Parasitic, and Allergenic Products, Office of Vaccines Research and Review, Center for Biologics Evaluation and Research, Food and Drug Agency, Silver Spring, MD 20993, USA; tod.merkel@fda.hhs.gov; 5 Department of Biostatistics, University of Michigan, Ann Arbor, MI 48105, USA; tombraun@umich.edu

**Keywords:** anthrax, virus-like particle, antibody, neutralization, lyophilization, epitope

## Abstract

Anthrax remains a formidable bioterrorism threat for which new, optimized and thermostable vaccines are needed. We previously demonstrated that five immunizations of rabbits with a multiple-antigenic-peptide (MAP) vaccine in either Freund’s adjuvant or human-use adjuvants can elicit antibody (Ab) against the loop-neutralizing determinant (LND), a cryptic neutralizing epitope in the 2β2-2β3 loop of protective antigen from *Bacillus anthracis* (*B. anthracis*), which mediates complete protection of rabbits from inhalation spore challenge with the *B. anthracis* Ames strain. To develop a more immunogenic vaccine, we molecularly constructed a virus-like particle (VLP) vaccine, comprising the Woodchuck hepatitis core antigen capsid (WHcAg) displaying 240 copies of the LND epitope on each nanoparticle. Initial studies showed that the LND-VLP was immunogenic in rabbits following two immunizations, and passive transfer of the rabbit sera into A/J mice conferred complete protection from aerosol challenge with *B. anthracis*. Further optimization of the vaccine revealed that the lyophilized LND-VLP vaccine was capable of eliciting highly protective levels of neutralizing antibody with two immunizations, and in some rabbits, a single immunization, using human-use adjuvants. A lyophilized LND-VLP nanoparticle vaccine may be an effective stand-alone vaccine or may complement PA-based vaccines as a future pre- or post-exposure vaccine for anthrax.

## 1. Introduction

While almost two and half decades have passed since spores of *Bacillus anthracis* (*B. anthracis*) were sent through the U.S. mail, resulting in five fatalities, inhalation anthrax still remains a serious bioterrorist threat. The relative simplicity of producing weapons-grade anthrax spores at scale and the extremely high lethality associated with exposure to such spores in unvaccinated individuals make the necessity for preparedness particularly critical [1,2].

Inhalation anthrax is highly lethal, with the morbidity and mortality primarily resulting from the production of two toxins, lethal toxin (LeTx) and edema toxin. These are A-B toxins, with lethal factor and edema factor representing the active components and protective antigen (PA) the cell-binding moiety [3,4,5]. Humoral immunity to PA successfully mediates protection from spore challenges in animal models of inhalation anthrax, and the levels of protection are directly correlated with the production of PA-specific antibodies (Abs) capable of neutralizing LeTx in the toxin neutralization assay (TNA) [6,7,8,9,10,11,12].

There have been continued evaluation and optimization of the anthrax vaccine currently approved in the U.S., BioThrax^®^ (Emergent BioSolutions, Gaithersburg, MD, USA), which is a culture filtrate from a toxigenic, non-encapsulated strain of *B. anthracis*, adsorbed to an Alum adjuvant [13]. PA is the major immunogenic component of BioThrax^®^, and immunization elicits neutralizing Ab which mediates protection from *B. anthracis* spore challenge in experimental animal models of anthrax [8,14]. Biothrax^®^ is currently administered as five immunizations over 18 months with a requirement for yearly boosting to maintain Ab titers [13]. More recently, a new vaccine, Cyfendus^®^, approved for use exclusively in post-exposure scenarios along with antibiotics, has combined the adjuvant CpG with Biothrax^®^ in a short-course, three-dose regimen for the elicitation of accelerated immune responses [15].

Ongoing efforts continue to be directed toward the development of new and stable alternative prophylactic and therapeutic vaccines for anthrax [2,16,17,18,19]. These efforts have been undertaken for manifold reasons. First, though the standard prophylactic protocol for Biothrax^®^ has been streamlined to five immunizations over 18 months with yearly booster immunizations [20], it still remains a challenging immunization protocol which can reduce compliance. Second, immunization is frequently accompanied by significant reactogenicity including localized erythema, swelling and pain, as well as systemic symptoms including fever, fatigue, headaches and myalgias [2,21,22]. Third, though efforts are underway to develop lyophilized recombinant PA for potential use in anthrax vaccines [23], the currently licensed vaccine requires a cold-chain, and must be replaced at regular intervals in the strategic national stockpile owing to well-described chemical alterations in PA during storage which reduce the immunogenicity of the vaccine [24,25,26,27]. The need for replacement of existing vaccine stocks, in turn, necessitates significant costs.

We have previously shown that immunization with epitope-focused immunogens in the form of either multiple antigenic peptides (MAPs) or a recombinant protein can elicit Ab specific for a linear determinant in the 2β2-2β3 loop of PA, which can mediate protection of rabbits from inhalation spore challenge with the *B. anthracis* Ames strain [28,29,30,31]. This protective neutralizing epitope, referred to as the loop-neutralizing determinant (LND), is found within a region of PA critical to the translocation of edema and lethal factor into cells to form edema toxin and LeTx, respectively, that contains the chymotrypsin cleavage site [3,4,5]. Mutagenesis of the sequence in the LND-binding epitope has been shown to negate the toxicity of LeTx, thereby rendering this neutralizing epitope relatively resistant to potential malicious re-engineering in a manner designed to circumvent the effectiveness of neutralizing antibody [32,33,34]. Indeed, directed mutations and deletions at Phe313-Phe314 in the LND epitope sequence have been employed to detoxify PA as a basis for new PA-based anthrax vaccines, including a recombinant PA-based anthrax vaccine under study for use in South Korea [35,36,37].

A significant body of data shows that antibody to the LND does not appear to be elicited through immunization with PA. No significant LND-reactive Ab was identified in the sera from rabbits and non-human primates immunized with PA, nor was significant Ab detected in the sera from a cohort of adults who were immunized with AVA (Biothrax^®^) in a clinical trial [28,30,38]. Since LND specificity is therefore non-overlapping with the neutralizing antibody specificities produced through immunization with Biothrax^®^ or other PA-based vaccines, the elicitation of this specificity through vaccination could potentially contribute to the neutralizing Ab repertoire elicited by existing PA-based anthrax vaccines.

While prior studies have demonstrated the protective efficacy in rabbits of vaccination with MAPs or a recombinant protein displaying the LND epitope, the vaccination protocols employed have all required five immunizations, whether using Freund’s adjuvant or using human-use adjuvants [29,30,31]. In an effort to develop a more immunogenic LND vaccine to elicit protective immunity in experimental animal models of anthrax with two or fewer doses, we molecularly constructed a virus-like particle (VLP) vaccine using the Woodchuck hepatitis core antigen capsid (WHcAg) displaying the LND epitope, and evaluated this prototype vaccine in mice and rabbits. These VLPs are highly immunogenic, self-assembling nanoparticles comprising 240 capsid protein monomers, each displaying the genetically fused LND epitope in a matrix array on the surface of the nanoparticle, and are highly effective for the elicitation of Ab. While they are very immunogenic using a wide array of adjuvants, including human-use adjuvants, and based on a viral protein sequence, VLPs are non-infectious, have an excellent safety profile equivalent to other recombinant proteins and are highly resistant to thermal breakdown [39].

## 2. Materials and Methods

### 2.1. Construction, Purification and Characterization of Recombinant WHcAg LND-VLPs

VLPs were constructed based on the full-length WHcAg (accession M18752) gene sequence codon-optimized for *E. coli* expression inserted into a pUC19 vector in place of the multiple cloning site. The LND epitope (single-letter a.a. code: GNAEVHASFFDIGGS), was engineered to be encoded either at amino acid 74 or between amino acids 78 and 79 of the WHcAg core protein gene [40]. The 74 insertion utilized a native SacI restriction site, and the 78/79 insertion relied on engineered restriction sites that added a Gly-Ile-Leu linker on the N-terminal side and a Leu linker on the C-terminal side of the inserted epitope. WHcAg constructs were transformed into DH5alpha-competent *E. coli*, grown in Terrific Broth and lysed by passage through an EmulsiFlex-C3 (Avestin, Ottawa, ON, Canada). The lysate was then heated to 65 °C for approximately 10 min, which precipitates unwanted proteins but leaves the highly thermostable VLP intact. The lysate was then clarified by centrifugation, and the WHcAg particles were selectively precipitated by the addition of solid ammonium sulfate to approximately 35% saturation (208 g/L). Precipitated VLPs were redissolved in minimum buffer (10 mM Tris, pH 8), and diafiltered with five volume exchanges of final formulation buffer in a hollow fiber cartridge with a 750 K molecular weight cutoff (WaterSep BioSeparations, Marlborough, MA, USA). The final formulation buffer was 20 mM Tris, pH8, 100 mM NaCl, 5 mM EDTA and 3% trehalose. The purified VLPs were 0.2 μm sterile-filtered, characterized and aliquoted. Characterization included native agarose gel electrophoresis, which allows visualization of the >50,000 kDa/>40 nanometer nanoparticles, and sodium dodecyl sulfate polyacylamide electrophoresis under reducing conditions for visualization of the approximately 24 kDa monomer. Further analysis included transmission electron microscopy (TEM) performed essentially as described [40,41]. The size and size distribution of the VLPs were determined by dynamic light scattering on a Zetasizer Nano ZS instrument (Malvern Panalytical Ltd., Malvern, UK) [42]. The antigenicity of the two LND-VLPs which were molecularly constructed was confirmed by the ELISA using high-titer rabbit antisera specific for the LND-MAP immunogen (Supplementary Figure S2). For some immunization studies, the purified LND-VLP was lyophilized, stored at 4C and reconstituted in molecular biology-grade water prior to use.

### 2.2. Animals and Vaccinations

For mouse experiments, F1 (B10 × B10.S) mice were bred and acquired from the Vaccine Research Institute of San Diego. BALB/c and CD1 mice were purchased from Jackson Labs (Jackson Labs, Bar Harbor, ME, USA). Mice were immunized i.p. with 20 μgs of the VLP in a water-in-oil emulsion with incomplete Freund’s adjuvant (IFA) on day 0, and then boosted one time 5–8 weeks post priming immunization. Mice were bled at week 11 post-priming immunization for analysis of sera by the ELISA and in the TNA. For the rabbit experiment employing IFA, female New Zealand white rabbits (Covance Research Products, Denver, PA, USA) weighing approximately 2.5 kg were immunized on day 0 with 250 μg of a VLP in an emulsion with IFA and boosted with 125 μg at weeks 5 and 12, also using IFA. For studies using human-use adjuvants, rabbits were primed s.c. with a range of 150–450 μg of VLPs mixed 1:1 (vol:vol) with Alhydrogel 2% (InvivoGen, San Diego, CA, USA) containing 100 μg of monophosphoryl lipid A (MPLA, Avanti Polar Lipids, Birmingham, AL, USA). For most experiments, except where noted, rabbits were boosted at 5 weeks post-priming immunization with a range of 75–225 μg of a VLP mixed with the same Alum/MPLA adjuvant combination. For procurement of antisera, rabbits were bled at varying time points as outlined in the figures and figure legends. All mice and rabbits were cared for in accordance with the standards of the Association for Assessment and Accreditation of Laboratory Animal Care, and all protocols were approved by the Institutional Animal Care and Use Committees of Explora BioLabs (approved protocol EB11-001) (Jackson Labs, Bar Harbor, ME, USA), the FDA (approved protocol #200413) and Covance Research Products (IACUC approval 19 March 2019) (Denver, PA, USA).

### 2.3. Enzyme-Linked Immunosorbent Assay

Antibody responses were assessed by the ELISA essentially as described [28]. Antibody titers were determined from serial two-fold dilutions of serum and represent the reciprocal dilution at the EC50 established using nonlinear regression to fit a variable-slope sigmoidal equation to the serial dilution data using Prism 10.0 (GraphPad Software, Inc., San Diego, CA, USA).

### 2.4. Toxin Neutralization Assay

The ability of antibody to block LeTx cytotoxicity in vitro was assessed using the RAW264.7 cell line (American Type Culture Collection, Manassas, VA, USA) as described [43]. For neutralization studies, approximately 120 ng/mL of PA (PA 171E, List Labs, Campbell, CA, USA) and 25 ng LF (LF169L, List Labs, Campbell, CA, USA) were used in the TNA. Contemporaneous toxin dose 50% (TD50) was determined for PA and LF with each assay to validate that the toxin concentrations used in each TNA were sufficient for greater than 90% cytotoxicity (4 TD50s). The reciprocal of the effective dilution (ED) protecting 50% of the cells from cytotoxicity (ED50) [44] was determined for each serum by using nonlinear regression to fit a variable-slope sigmoidal equation to the serial dilution data set using Prism 10.0. TNA results are displayed as NF50s (NF50 = ED50 sample/ED50 AVR801), which normalize the TNA results to AVR801 (human anti-AVA reference serum AVR801, Lot 2, BEI Resources, Manassas, VA, USA) [14]. The standard TNA has a lower limit of quantification of 16. Samples with a TNA below this limit were assigned a value of 8 (0.01 NF50).

### 2.5. Aerosol Spore Challenge

Male and female A/J mice (n = 8) approximately 6–12 weeks of age (Jackson Laboratory, Bar Harbor, ME, USA) were used for the aerosol challenge. For challenge, mice were exposed to aerosolized spores of *B. anthracis* strain 7702 for 90 min using a nose-only apparatus (CH Technologies, Westwood, NJ, USA) as described previously [45]. The spore inoculum for each challenge contained approximately 15 mLs of 5 × 10^9^ spores/mL in distilled H_2_O with 0.01% Tween 80. Following challenge on day 0, clinical observations were made for 12 days and moribund animals were euthanized. Deaths were recorded on the day the animal was found dead or was euthanized. Mice were housed and maintained at the Center for Biologics and Evaluation Research animal facility and were cared for in accordance with the standards of the Association for Assessment and Accreditation of Laboratory Animal Care and protocols approved by the Institutional Animal Care and Use Committees at the FDA.

### 2.6. Statistical Analysis

The Mann–Whitney and Kruskal–Wallis tests were used for comparing titers between two groups and more than two groups, respectively. The Kaplan–Meier method was used to plot survival data, and differences in survival were compared using the Mantel–Cox log-rank test. For all statistical analysis, a *p* value of <0.05 was considered significant. All statistical analysis was performed using GraphPad Prism version 10 (GraphPad Software, San Diego, CA, USA).

## 3. Results

### 3.1. Molecular Construction and Immunogenicity of VLPs Displaying the LND

**Figure 1:**
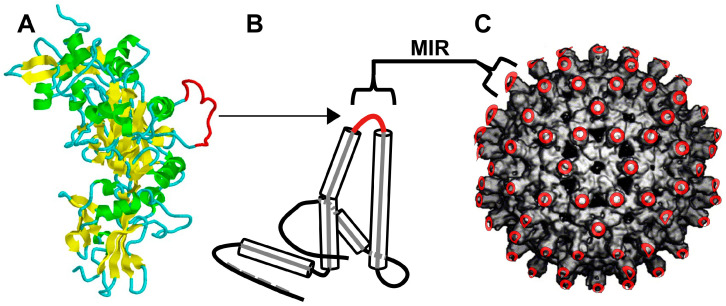
Molecular construction of the LND-VLP. Shown in (**A**) is a protein structural model of PA83 based on the 1TZO crystal structure, with the deduced location of the LND sequence (a.a. 305–319; single-letter a.a. code: GNAEVHASFFDIGGS) in PA83 highlighted in red. In (**B**) is a diagrammatic representation of the protein structural model of the WHcAg monomer, with alpha-helices depicted as cylinders and the insertion sites for the LND epitope within the major immunogenic region (MIR) of the WHcAg shown in red. Each WHcAg monomer forms dimers which then self-assemble into the nanoparticle (**C**) comprising 240 monomers, each displaying the MIR sequences (location depicted in red). The image in panel (**A**) was created in RasMol based on the 1TZO crystal structure of PA.

We previously demonstrated that the LND sequence, located in the 2β2-2β3 loop of PA (Figure 1A), represented a potent neutralizing epitope, and antibody elicited to this sequence displayed on MAPs or in a recombinant protein could elicit high-titer neutralizing antibody capable of mediating protective efficacy in rabbits upon aerosol spore challenge with the *B. anthracis* Ames strain [29,30,31].

**Figure 2:**
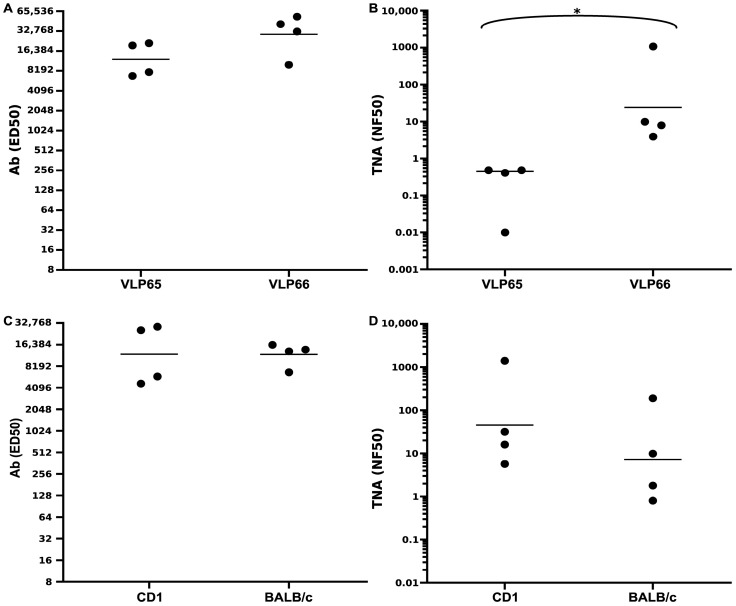
Immunogenicity of the LND-VLPs in mice. Antibody (**A**) and TNA (**B**) titers from week-11 sera from groups (n = 4) of F1 mice immunized two times with either the VLP65 or VLP66 nanoparticles in an emulsion with IFA. * GMT neutralization (NF50s) in the sera of mice immunized with the VLP66 was significantly higher than the GMT for mice immunized with the VLP65 (*p* = 0.029, Mann–Whitney test). Shown in (**C**,**D**) are Ab and TNA titers from 11-week sera from groups (n = 4) of CD1 and BALB/c mice immunized two times with the VLP66 in IFA. In all figures, horizontal lines are geometric means.

The immunization protocols employed in these rabbit studies all utilized five immunizations, regardless of whether Freund’s adjuvant or human-use adjuvants were used. To develop an LND vaccine capable of eliciting protective levels of neutralizing Ab with fewer immunizations using human-use adjuvants, we molecularly constructed two distinct VLPs, referred to as VLP65 and VLP66, which display the LND sequence at two alternative sites, positions 74 and 78, respectively, within the major immunodominant region (MIR) of the WHcAg monomer (Figure 1B). In solution, the monomers form strong dimers, and then self-assemble into nanoparticles (Figure 1C). Following the purification of the VLPs, we coated the VLPs on 96-well polystyrene plates and confirmed their immunoreactivity with LND-MAP-specific rabbit antisera by the ELISA (Supplementary Figure S2). We then assessed the immunogenicity of the VLPs in F1 mice, which our prior work has shown are excellent screening haplotypes for the WHcAg VLPs [46,47]. Groups of F1 mice (n = 4) were immunized with 20 μg of the respective VLPs in an emulsion with IFA on day 0 and were boosted with the same dose at week 8. Three weeks after the booster immunization (week 11), all mice were bled and sera were analyzed by the ELISA. As shown in Figure 2A, both VLPs elicited high-titer LND-specific Ab when assessed by the ELISA. When evaluated in the TNA, however, sera from mice immunized with the VLP66 demonstrated significantly (*p* = 0.029) higher toxin neutralization titers compared to sera from mice immunized with the VLP65 (Figure 2B). We therefore considered the VLP66, henceforth referred to as the LND-VLP, as the lead LND nanoparticle and proceeded to perform additional immunogenicity testing in two additional strains of mice. Groups of BALB/c and CD1 mice (n = 4) were immunized twice with the LND-VLP in an emulsion with IFA, and 11-week sera were analyzed by the ELISA and in the TNA. In both haplotypes, the LND-VLP elicited LND-specific Ab which was immunoreactive with immobilized PA83 in the ELISA (Figure 2C), and demonstrated neutralization in the TNA (Figure 2D).

### 3.2. Immunogenicity of the LND-VLP in Rabbits

**Figure 3:**
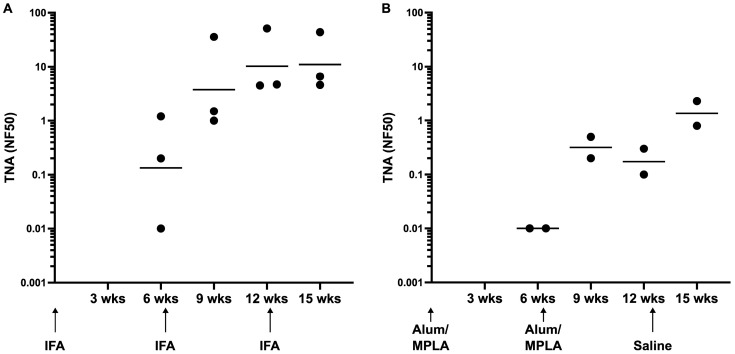
Immunogenicity of the LND-VLP in rabbits. Shown in (**A**) are TNA titers from sera obtained at 6, 9, 12 and 15 weeks post-priming immunizations from a group of rabbits (n = 3) immunized three times with the LND-VLP in an emulsion with IFA, or in (**B**) a group of rabbits (n = 2) immunized twice with the LND-VLP using Alum/MPLA and given a third injection with the LND-VLP in saline only. Arrows denote the immunization time points for both groups at priming (time 0) and at 6 and 12 weeks post-priming immunizations. Bleeds obtained at 6 and 12 weeks were procured prior to immunizations. Horizontal lines are geometric means.

As rabbits and non-human primates (NHPs) are the two most accepted animal models for evaluating anthrax vaccines [7], we next evaluated the immunogenicity of the LND-VLP in rabbits. While WHc-VLPs are highly immunogenic when formulated as emulsions, most commonly employing water-in-oil emulsions like IFA or ISA720, or oil-in-water emulsions like MF59, we ultimately planned to employ either adjuvants currently licensed for human use or ones undergoing testing for use in humans, for future testing of the LND-VLP in larger animal models. In an initial exploratory experiment, we immunized one group of rabbits (n = 3) with the LND-VLP in an emulsion with IFA, and a second group of rabbits (n = 2) with the LND-VLP adsorbed to Alhydrogel plus the toll receptor 4 agonist MPLA. Both groups of rabbits were boosted at week 6 using the respective adjuvants employed for the priming immunizations, and a second boost was administered at week 12 using either IFA for the LND-VLP-IFA group, or saline only for the group receiving the LND-VLP using Alum/MPLA. As shown in Figure 3A, the LND-VLP was immunogenic in both adjuvants and elicited neutralizing Ab in all rabbits after two immunizations. A second booster immunization (third injection) employing IFA had no detectable effect on neutralization titers in the IFA group, but led to a marked increase in neutralization in both rabbits in the Alum/MPLA group which received their final boost in saline only (Figure 3B).

### 3.3. Efficacy of the LND-VLP-Specific Rabbit Antisera for Passive Protection of Mice from Anthrax Spore Inhalation Challenge

To establish proof of concept that the antibody elicited to the LND-VLP in rabbits has the capability for protection of rabbits from spore challenge, as was demonstrated previously with the LND in MAP and recombinant forms [29,30,31], without performing an expensive and resource-intensive anthrax inhalation spore challenge in rabbits, we assessed the capacity and relative potency of passively transferred LND-VLP-specific rabbit antisera to protect mice from a lethal aerosol spore challenge with the *B. anthracis* Sterne strain using a well-established mouse inhalation model [45]. Additionally, we benchmarked the relative protective potency of the LND-VLP antisera to pre-challenge antisera from a rabbit which had the highest neutralization among a group of seven rabbits, all immunized with an LND-MAP vaccine and subsequently protected from a 200-LD50-targeted aerosol challenge with the *B. anthracis* Ames strain in a prior study [30].

**Figure 4:**
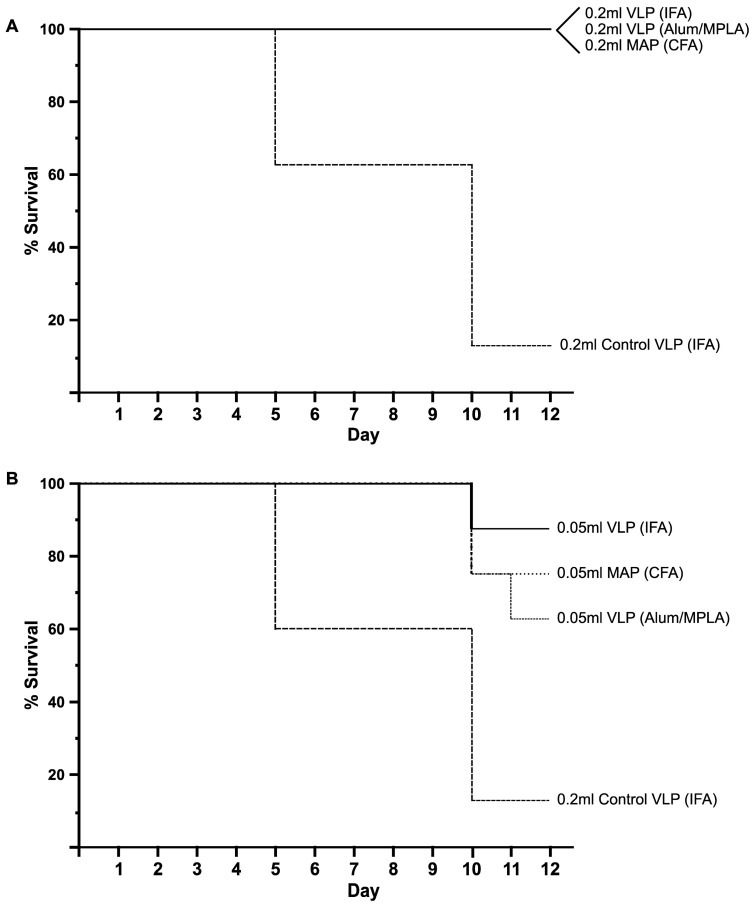
Passive protection of mice from anthrax aerosol spore challenge using rabbit antisera to the LND-VLP. Kaplan–Meier survival curves from groups of A/J mice which were passively immunized with rabbit antisera prior to aerosol challenge with 5 LD50s of *B. anthracis* Sterne strain as described in methods. One day prior to aerosol challenge, groups of mice (n = 8) were passively immunized with a single medium (0.2 mL) or low (0.05 mL) dose of week-15 antisera from a rabbit immunized three times with the LND-VLP in IFA or the LND-VLP using Alum/MPLA/saline. The positive control sera (MAP-CFA/IFA) was obtained from a rabbit at approximately 10 weeks post-priming immunization which had been immunized 5 times in two-week intervals with an LND-MAP vaccine in an emulsion with CFA/IFA prior to undergoing and surviving a 200 LD50-targeted *Bacillus anthracis* Ames strain aerosol spore challenge in a prior study [30]. A single negative control group of mice (n = 8) received a medium dose (0.2 mL) of antisera from a rabbit immunized three times with an irrelevant VLP in Alum/MPLA. In (**A**), 8/8 (100%) mice in all of the medium-dose LND antisera groups survived, while 1/8 (12.5%) mice receiving the control sera survived to day 14 (*p* = 0.00001, log rank compared to negative control). In (**B**), 7/8 mice (87.5%) receiving the low-dose sera from a rabbit immunized with the LND-VLP/IFA survived, while 5/8 mice (62.5%) and 6/8 (75%) mice receiving the low-dose sera from a rabbit immunized with the LND-VLP/Alum/MPLA/saline or the positive control LND-MAP sera survived to day 14, respectively (*p* = 0.0001 log rank for all low-dose groups compared to control).

One day prior to aerosol challenge, separate groups of A/J mice (n = 8) were passively immunized i.p. with a medium (0.2 mL) or low (0.05 mL) dose of antisera from the rabbits with the highest neutralization at 15 weeks from the LND-VLP-IFA group or the LND-VLP–Alhydrogel/MPLA/saline group. Positive control groups were passively immunized with either a medium or low dose of week-10 pre-challenge antisera, obtained from a single rabbit with the highest neutralization titer among a group of rabbits immunized five times with an LND-MAP in Freund’s adjuvant, all of which were subsequently protected from a 200 LD50-targeted aerosol spore challenge with the *B. anthracis* Ames strain [30]. A single negative control group was passively immunized with a medium dose (0.2 mL) of rabbit antisera elicited to an irrelevant VLP in IFA. Twenty four hours later, all mice underwent aerosol challenge with approximately 5 LD50s of the *B. anthracis* Sterne strain, essentially as described [45]. As shown in Figure 4A, both groups of mice passively immunized with the medium doses of LND-VLP antisera, as well as mice administered the medium dose of positive control LND-MAP antisera, were completely protected from aerosol challenge, while seven out of eight negative control mice died by day 10 post-challenge (*p* = 0.00001, log rank compared to negative control). The protective efficacy of the low-dose rabbit antisera from the LND-VLP/IFA (7/8 protected; 87.5% survival), LND-VLP/Alhydrogel/MPLA (5/8 protected; 62.5% survival) and the positive control LND-MAP (6/8 protected; 75% survival) was significantly different compared to the negative control (*p* = 0.0001, log rank compared to control) but was not significantly different among the low-dose LND-specific sera groups (Figure 4B). Since the positive control sera was from the rabbit with the highest pre-challenge neutralization titer among a group of rabbits all protected from subsequent Ames strain inhalation spore challenge, the results suggest that the LND-VLP vaccine has potential to be efficacious for protecting rabbits from aerosol challenge with two immunizations in human-use adjuvants.

### 3.4. Optimization of Immunization in Rabbits with the LND-VLP

**Figure 5:**
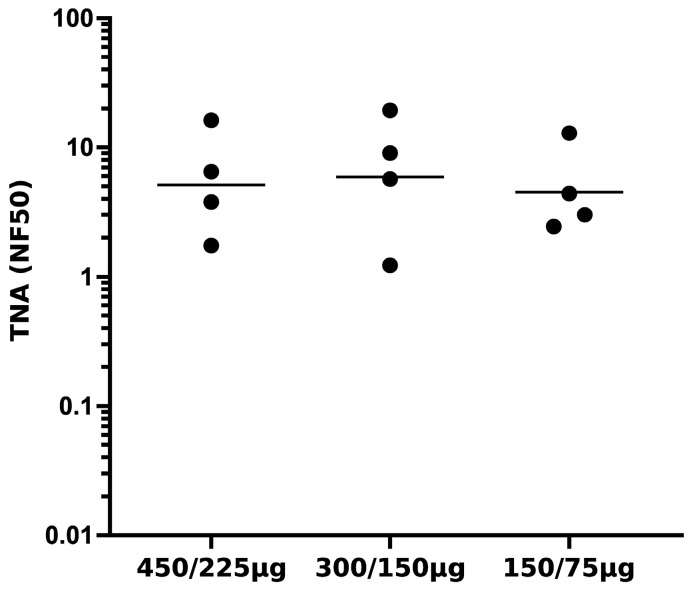
Dose response to the LND-VLP in rabbits. Shown are TNA titers from sera obtained at week 8 from groups of rabbits (n = 4) immunized twice (wk 0, wk5) using a combined s.c/i.m. route with the indicated doses (priming/boosting) of the LND-VLP using Alum/MPLA adjuvant. Horizontal lines represent geometric means.

Having demonstrated that rabbit antisera raised to the LND-VLP using Alum/MPLA/saline can confer passive protection of mice from *B. anthracis* Sterne aerosol challenge, we sought to optimize the immunization protocols for eliciting high-titer neutralizing antibody in rabbits using two doses of the LND-VLP with the Alum/MPLA adjuvant. In a series of immunogenicity experiments in rabbits, we determined that a combined s.c. and i.m. route of immunization was most effective for eliciting high-titer neutralizing Ab in rabbits compared to either exclusively s.c or exclusively i.m routes. Next, a dose–response study in rabbits revealed that two doses of the LND-VLP administered using the combined sc./i.m route with Alum/MPLA were equivalently immunogenic in a range of priming doses from 150 μg to 450 μg, with a single booster dose of one-half the priming dose given at week 5 (Figure 5).

### 3.5. Immunogenicity of the Lyophilized LND-VLP in Rabbits

**Figure 6:**
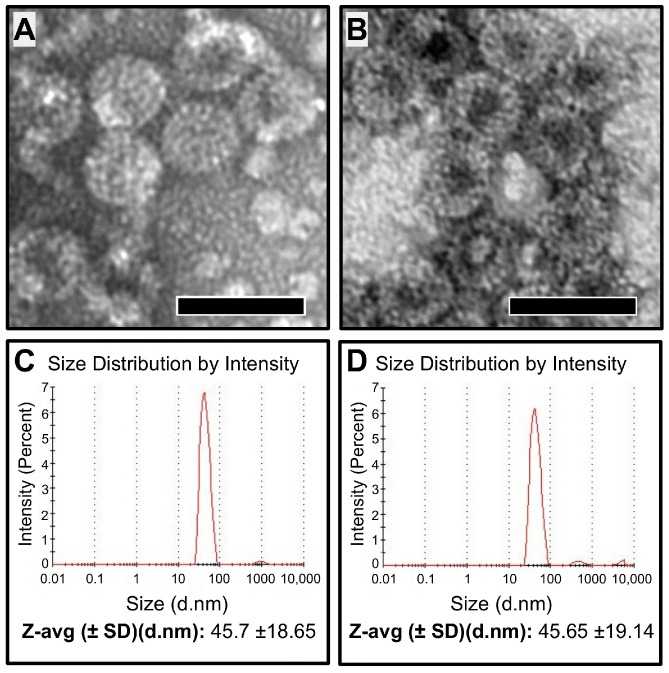
Transmission electron microscopy and dynamic light scattering of the lyophilized and soluble forms of the LND-VLP. Transmission electron microscopy of the non-lyophilized (**A**) and lyophilized and reconstituted LND-VLP (**B**). Magnification = 40,000×. Scale bar = 50 nm. In (**C**,**D**) are shown the results from dynamic light scattering sizing for the non-lyophilized (**C**) and lyophilized/reconstituted (**D**) LND-VLP nanoparticles.

As mentioned, the current anthrax vaccine approved for use in the U.S., Biothrax^®^, and the vaccine approved for use in the U.K., AVP, both require refrigeration for storage under current guidelines and cannot be stored lyophilized [48]. Our prior work on WHc-based VLPs for malaria demonstrated that the VLPs maintain their molecular structure and immunogenicity following lyophilization and reconstitution. We also found this to be the case with the LND-VLPs, which, following lyophilization and reconstitution, form 40–50-nanometer icosahedral particles, by transmission electron microscopy (Figure 6B), which are nearly indistinguishable from the LND-VLP which did not undergo lyophilization and reconstitution (Figure 6A). The size distributions of the LND-VLP nanoparticles that were lyophilized and reconstituted (Figure 6D) were also determined to be the same as the size distributions of the LND-VLP that did not undergo lyophilization (Figure 6C) as deduced through dynamic light scattering, with average particle diameters of 45.65 and 45.7 nanometers, respectively.

**Figure 7:**
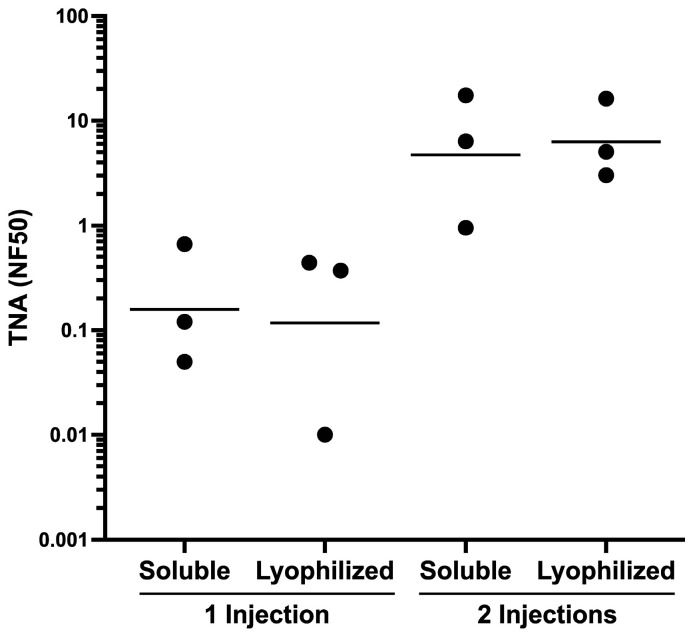
Immunogenicity in rabbits of the lyophilized and soluble forms of the LND-VLP. Groups of rabbits (n = 3) were immunized at week 0 and week 5 with either the soluble (non-lyophilized) LND-VLP or the lyophilized and reconstituted LND-VLP using Alum/MPLA adjuvant. Serum neutralization titers were determined after one injection (week-5 sera, prior to boost) or two injections (week-8 sera). Horizontal lines represent geometric means.

To compare the immunogenicity of the lyophilized LND-VLP with that of the non-lyophilized LND-VLP, separate groups of rabbits (n = 3) were primed with 400 μg of either the lyophilized/reconstituted LND-VLP or the non-lyophilized LND-VLP mixed with Alhydrogel/MPLA using a combined s.c./ i.m. route. Five weeks later, all rabbits were boosted s.c/i.m with 200 μg of the respective VLPs in Alum/MPLA. Rabbits were bled at week 5 prior to the booster immunization and at week 8, 3 weeks after the booster immunizations, to assess the sera for neutralization. As shown in Figure 7, sera from rabbits immunized with the lyophilized/reconstituted and non-lyophilized LND-VLP elicited high levels of neutralization after two immunizations, and there were no significant differences between their respective neutralization titers.

### 3.6. Performance of a Rabbit Pre-Aerosol Challenge Immunization Regimen

**Figure 8:**
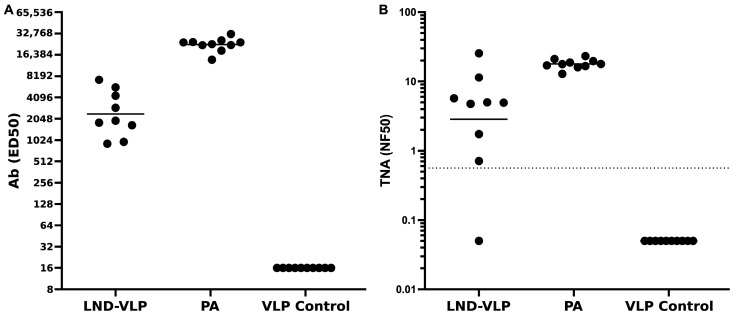
Antibody and TNA titers in groups of rabbits immunized using a pre-aerosol spore challenge immunization regimen. Groups of rabbits (n = 10) were immunized with either the lyophilized and reconstituted LND-VLP in Alum/MPLA, PA83 in Alum (positive control) or an irrelevant VLP (negative control) in Alum/MPLA at wk 0. The PA control group was boosted at week 4 with PA in Alum, and both VLP groups were boosted at week 5 with their respective immunogens in Alum/MPLA. Sera were obtained at 9 weeks post-priming for analysis of Ab (**A**) and TNA (**B**) titers at a time point designed to simulate when an anthrax spore aerosol challenge would typically occur. Horizontal lines represent geometric means. The dotted line in (**B**) represents an NF50 of 0.56, which has been shown to predict a 70% likelihood of protection in rabbits, and an 88% probability of protection of NHPs [14].

In lieu of performing an expensive, resource-intensive rabbit challenge at this point in the development of the LND-VLP vaccine, which was out of scope for these studies, we opted to perform only the immunization component that would precede an anthrax spore aerosol challenge, and rely on well-established, highly predictive probability models for analysis of the immunization data. These probability models use in vitro serum neutralization levels as surrogates for predicting survival in both the rabbit and NHP anthrax models [14,49]. Groups of NZW rabbits (n = 10; 5 female, 5 male) were immunized twice with either the lyophilized/reconstituted LND-VLP in Alum/MPLA, PA83 in Alum (positive control) or an irrelevant VLP in Alum/MPLA (negative control). One rabbit in the LND-VLP group died from causes unrelated to immunization during the course of the study. All rabbits were boosted once with their respective immunogen/adjuvant mixtures, at day 28 for the PA83 group, and at day 35 for the VLP groups. Serum was obtained eight weeks post-priming immunization for all groups for assessment of Ab and neutralization titers. As shown in Figure 8A, all rabbits immunized with the LND-VLP or PA83 elicited Ab reactive with PA in the ELISA. Group-specific geometric mean neutralization titers (NF50) were 2.8 and 17.9 for the LND-VLP and PA83 groups, respectively. With the use of an NF50 level of 0.56 (Figure 8B, dotted line), which is associated with a 70% and 88% probability of survival for rabbits and NHPs, respectively, from high-dose aerosol Ames strain challenge [14], the LND-VLP, PA83 and negative control groups would be predicted to have 8/9 (89%), 10/10 (100%) and 0/10 (0%) survival if the rabbits had undergone an actual Ames strain aerosol spore challenge at approximately 9–10 weeks post-priming immunizations.

## 4. Discussion

Our prior work demonstrated that five immunizations of rabbits using Freund’s or human-use adjuvants with either MAPs or a recombinant protein displaying the LND peptide epitope were capable of completely protecting rabbits from high-dose Ames strain aerosol spore challenge [29,30,31]. In the current study, we evaluated whether displaying the LND epitope on the WHcAg VLP would improve the immunogenicity of a prototype LND vaccine and reduce the number of immunizations required to achieve protective levels of neutralization in rabbits, one of the two commonly accepted animal models, along with NHPs, for evaluating prototype vaccines for anthrax. Immunization in three strains of mice, including the non-inbred CD1 strain, demonstrated that the LND-VLP was effectively displayed on the VLP nanoparticle and was capable of eliciting high-titer neutralization using water-in-oil emulsions. Subsequent work demonstrated that antisera from rabbits immunized with three doses of the LND-VLP in either IFA or Alum/MPLA were able to confer passive protection of mice from aerosol challenge with *B. anthracis*, and the relative potency of the antisera, benchmarked against sera from a rabbit which had the highest neutralization titer among seven rabbits all subsequently protected from a high-dose Ames strain challenge, suggested that the LND-VLP vaccine, once optimized, could likely elicit protection in rabbits using only two doses in Alum/MPLA. Indeed, optimization of the route of delivery of the vaccine, from either exclusively s.c. or exclusively i.m., to a combination of s.c. and i.m., led to the consistent elicitation in rabbits of supra-protective levels of neutralization using Alum/MPLA with geometric mean NF50s in the range of 4–5. This is substantially above the in vitro surrogate level of neutralization of 0.56 NF50s, which has been shown to have a 70% and 88% probability of predicting protection from anthrax spore inhalation challenge in rabbit and NHPs, respectively [14]. The improved immunogenicity using a combined s.c. and i.m. route of immunization may be related to a broader presentation of the LND-VLP to antigen-presenting cells. The particulate nature of VLPs in the size range of 40–50 nm is believed to contribute to the superior immunological properties associated with VLPs through facilitation of uptake by dendritic cells leading to processing and presentation by MHC Class II [50,51]. Our work has shown that the WHc VLPs contain numerous and variable sources of helper T cell epitopes in multiple inbred strains of mice [52,53], and we have not observed an Ab non-responder following vaccination of mice and outbred rabbits with WHc VLPs in disease models including malaria and RSV [39,46].

Importantly, the lyophilized and reconstituted LND-VLP was equivalently immunogenic compared to the LND-VLP which had not undergone lyophilization. The capability for lyophilization represents a highly desirable feature for anthrax vaccines, regardless of whether they are used in a prophylactic or post-exposure setting, as they require stockpiling in large quantities due to the low-probability but very-high-risk threat associated with the bioterrorist delivery of disseminated anthrax spores. Having established the ability to lyophilize the LND-VLP and reconstitute it without loss of immunogenicity, we plan to evaluate the stability of the LND-VLP and assess whether it will be able to bypass the need for a cold-chain. The necessity of a vaccine cold-chain in general, and specifically for anthrax vaccines, can engender significant costs both for replacement of existing stocks of vaccines, as currently occurs with Biothrax^®^, and for the logistics of administration of vaccines at the point of contact with vaccinees.

In lieu of a resource-intensive and expensive aerosol challenge, as has been performed previously with MAP and recombinant proteins displaying the LND epitope, we opted to perform only the immunization protocol that would occur prior to an Ames strain spore inhalation challenge in groups of rabbits, and use the highly predictive serum in vitro neutralization levels to assess the probability of protection from spore challenge. Our data indicated that 89% (8/9) of the rabbits immunized twice with the lyophilized LND-VLP in Alum/MPLA would have likely survived a 200 LD50-targeted spore challenge at approximately 8–9 weeks post priming immunization, while the positive control rabbits, immunized with PA in Alum, the commonly accepted adjuvant for evaluating PA in rabbits and NHPs, would have been 100% protected. While the overall titers of LND Ab elicited using the Alum/MPLA adjuvant were not extremely high, as might be expected had we employed IFA or other water-in-oil emulsions, the LND-specific Ab demonstrates high specific activity for achieving neutralization. This is consistent with our prior work with LND vaccines and may reflect the sensitivity to Ab-mediated neutralization of the LND epitope sequences, which encompass the chymotrypsin cleavage site in the 2β2-2β3 loop of PA. Across multiple prior Ames strain aerosol challenges, only a single rabbit out of 51 rabbits with any detectable LND-mediated neutralization succumbed to Ames strain spore challenges, and a number of these rabbits had NF50s appreciably below 0.56 [29,30,31]. Nevertheless, the challenges of eliciting high-titer Ab to recombinant proteins like VLPs while employing human-use adjuvants do highlight the need to test and develop more potent adjuvants for use with epitope-focused recombinant vaccines. The doses of VLPs employed in our studies ranged from priming doses of 400 μg to 150 μg, and our data shows that such doses were equivalently immunogenic. While these doses seem considerable, we did not observe any adverse effects in the immunized rabbits. In studies evaluating the immunogenicity of recombinant PA in rabbits, priming dose ranges from 250 μg to 100 μg have frequently been employed, and yet the approximate dose of PA in a human dose of Biothrax^®^ is only 50 μg [7]. We would anticipate a similar dose range of 50–100 μg for future analysis of the LND-VLP in NHPs.

While none of the rabbits immunized with the LND-VLP in the pre-aerosol challenge immunization regimen were Ab non-responders, one rabbit did not have detectable neutralization and may have succumbed during an actual anthrax inhalation spore challenge. Though the elicitation of LND-specific Ab without functional in vitro neutralization activity has been extremely uncommon in our work to date with LND-containing immunogens, it does highlight a potential limitation of an anthrax vaccine targeting only the LND. The single point of failure associated with only targeting a single epitope, despite that epitope reflecting a highly sensitive and potentially important target, likely necessitates the identification of additional epitopes in either PA or LF for use in combination with the LND-VLP as a potential prophylactic epitope-specific anthrax vaccine for general use. Nevertheless, the lack of Ab to the LND in vaccine sera from individuals immunized with PA vaccines, the apparent sensitivity of the LND sequences in PA for Ab-mediated neutralization of LeTx and the capability for lyophilization and reconstitution of the LND-VLP without loss of immunogenicity all encourage further development of this vaccine for use as an adjunct prophylactic or post-exposure vaccine for anthrax.

## Data Availability

The original contributions presented in this study are included in the article/Supplementary Material. Further inquiries can be directed to the corresponding author.

## Supplementary Materials

The following supporting information is available online at https://www.mdpi.com/article/10.3390/microorganisms13081878/s1. Figure S1: Fully uncropped fields for TEMs as captured for Figure 6A,B. Figure S2: Reactivity of anti-LND rabbit sera with VLPs 65 and 66 by ELISA.

## Abbreviations

The following abbreviations are used in this manuscript:

VLP	Virus-like particle
TNA	Toxin neutralization assay
LND	Loop-neutralizing determinant
MAP	Multiple antigenic peptide
LeTx	Lethal toxin
WHcAg	Woodchuck hepatitis core antigen
