# Supplementary material for: A Lyophilizable Nanoparticle Anthrax Vaccine Targeting the Loop-Neutralizing Determinant in Protective Antigen from Bacillus anthracis
Source: Microorganisms. 2025 Aug 12;13(8):1878. doi: 10.3390/microorganisms13081878 (PMC12388387; doi:10.3390/microorganisms13081878)
Supplement: Supplementary file 1 [file microorganisms-13-01878-s001.zip › Figure S1.pdf]

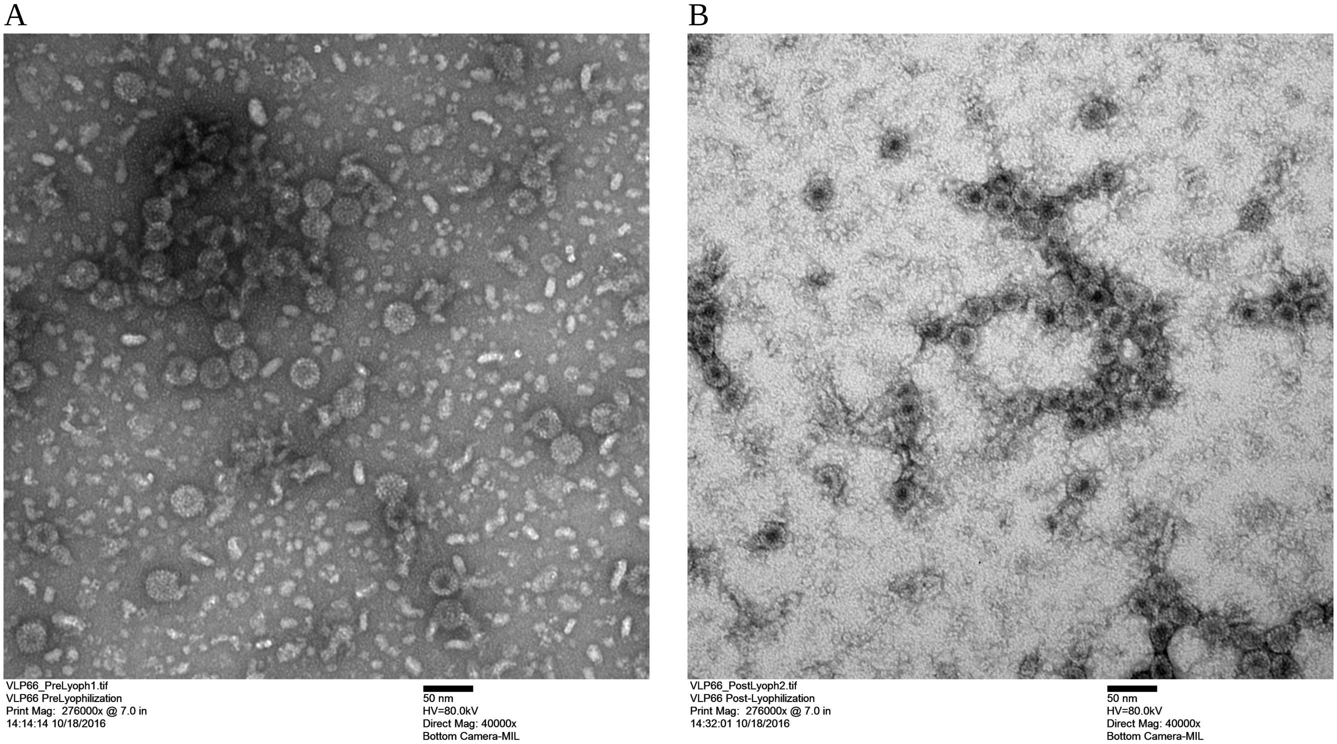

Figure S1. Uncropped transmission electron micrographs (TEM) from figure 6. A. Purified LND-VLP (VLP66) before lyophilization. B. LND-VLP (VLP66) after lyophilization and reconstitution. Details from the transmission microscope, including scale bar, are at the bottom of each uncropped image.
