# Supplementary material for: A Lyophilizable Nanoparticle Anthrax Vaccine Targeting the Loop-Neutralizing Determinant in Protective Antigen from Bacillus anthracis
Source: Microorganisms. 2025 Aug 12;13(8):1878. doi: 10.3390/microorganisms13081878 (PMC12388387; doi:10.3390/microorganisms13081878)
Supplement: Supplementary file 1 [file microorganisms-13-01878-s001.zip › Figure S2.pdf]

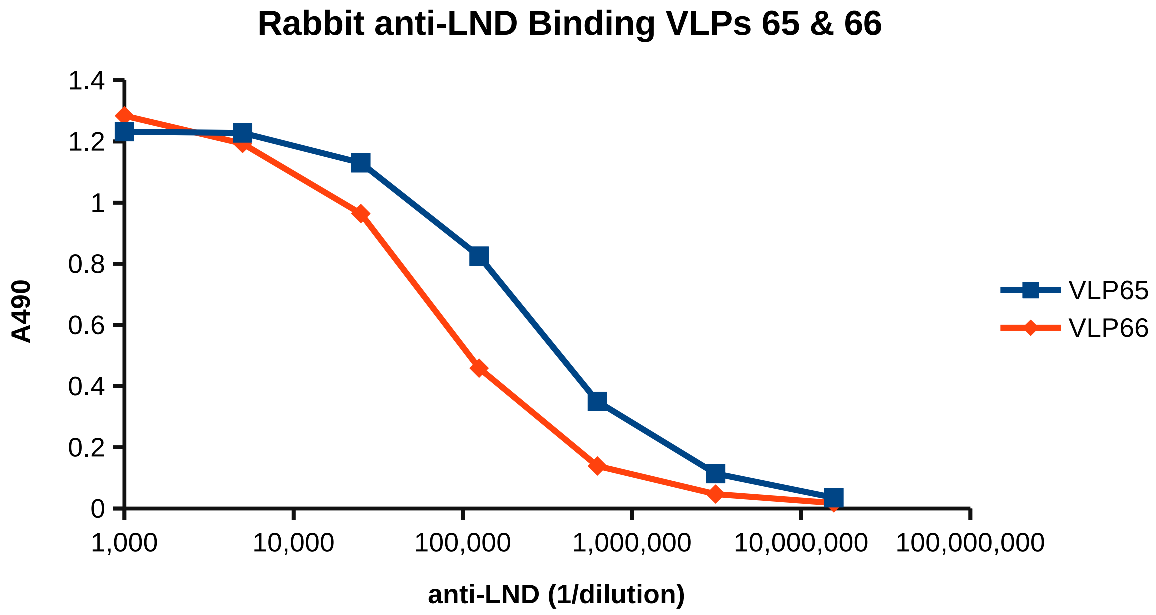

Figure S2. Reactivity of anti-LND rabbit sera with VLPs 65 and 66 by ELISA. Plates were coated with each VLP at 1μg/ml and detected by high titer rabbit sera elicited to an LND MAP.
